# Dietary intake and sleep in late childhood – do shorter children have distinct patterns?

**DOI:** 10.1038/s41390-024-03678-3

**Published:** 2024-11-06

**Authors:** Naama Fisch-Shvalb, Maya Zur, Liora Lazar, Raanan Shamir, Moshe Phillip, Michal Yackobovitch-Gavan

**Affiliations:** 1 Faculty of Medicine, Tel Aviv University, Tel Aviv, Israel; 2 The Jesse Z and Sara Lea Shafer Institute for Endocrinology and Diabetes, National Center for Childhood Diabetes, Schneider Children’s Medical Center of Israel, Petah Tikva, Israel; 3 Department of Epidemiology and Preventive Medicine, School of Public Health, Faculty of Medicine, Tel Aviv University, Tel Aviv, Israel; 4 Institute for Gastroenterology, Nutrition and Liver Diseases, Schneider Children’s Medical Center, Petah Tikva, Israel

## Abstract

**Background:**

The exceptional growth rate during adolescence demands increased dietary intake. We aimed to compare diet and lifestyle of pre-adolescents with height and weight below the 10^th^ percentile, with those of pre-adolescents of higher height and weight.

**Methods:**

This case-control study included healthy pre-pubertal girls (≥9 years) and boys (≥10 years). The case groups included 31 girls and 32 boys with height and weight <10th percentile, and weight percentile ≤height percentile. The control groups comprised 24 girls and 24 boys, with height ≥25th percentile, BMI 5th–85th percentiles. Participants completed 3-day food diaries and lifestyle-related questionnaires.

**Results:**

Energy intake/estimated-requirement and protein/body-weight were comparable in the case and control groups, both in boys and girls. In boys, fat (*P* = 0.050) and carbohydrate (*P* ≤ 0.001) intakes/body-weight were higher in the case group versus controls; and iron (*P* < 0.001), zinc (*P* = 0.005), vitamin A (*P* < 0.001), calcium (*P* = 0.005), and vitamin C (*P* = 0.034) consumption were lower. In girls, carbohydrate/body-weight was higher in the case group compared to controls (*P* = 0.007); micronutrient intake was comparable, and lower than recommended. Compared to controls, short and thin boys reported less sleep during weekdays (*P* < 0.001).

**Conclusions:**

Relatively short, thin pre-adolescents may have distinct dietary intake and sleeping patterns compared to taller peers, especially boys.

**Impact:**

- Nutrition is a key environmental determinant of childhood growth. Not much is known about late nutritional impact on growth in children whose anthropometric indices are below the 10th percentile.
- We compared food diaries and lifestyle questionnaires of pre-pubertal pre-adolescents with height <10th percentiles and weight≤height for age and sex, with those of peers with indices in higher percentiles.
- We found distinct differences in micronutrient intakes and sleep duration in the shorter boys, but no energy deficit in either sex.
- We suggest that diet and lifestyle parameters should be evaluated in children with relatively low anthropometric indices, to ensure optimal growth.

## Introduction

Nutrition is one of the most important environmental influences on childhood growth. In extreme conditions of malnutrition, linear growth is clearly compromised, as shown in numerous studies from low-income countries.^1^ Poor dietary quality and micronutrient deficiencies are also found in children and adolescents living in affluent societies,^2^ however, it is unclear to what extent this affects their linear growth. Otherwise healthy children with height below the 3rd percentile for age and sex, tend to be leaner than peers with normal height,^3^ and to have distinct eating patterns.^4,5^ In this population, suboptimal nutritional intake^4,6,7^ and micronutrient deficiencies^8^ have been described. Postulating that suboptimal nutrition may impact growth in children with even a mild compromise in height, we previously showed that this might be the case in young children with only relatively low height and weight-Z scores (height and weight <10th percentile for age and sex, and weight percentile ≤height percentile). These children, aged 3–9 years, consumed a poorer diet than their peers who were of higher anthropometric indices (height >25th percentile, and a body mass index (BMI) within the 5–85th percentile for age and sex).^6^ In the current study, we aimed to assess the dietary and lifestyle parameters of older children with similarly low height and weight-Z scores (referred to as “low height and weight”, LHW), compared to peers with height and weight in higher percentiles. The primary objective of this study was to compare the nutrient intakes of these groups, while our secondary goal was to compare their self-reported physical activity levels, and sleep patterns.

## Methods

A case-control study was performed at a Pediatric Endocrinology institute in a tertiary medical center between January 2017 and September 2019. Eligible participants for the case groups were healthy, pre-pubertal pre-adolescents with height and weight <10th percentile for age and sex, and weight percentile ≤height percentile according to the 2000 Centers for Disease Control (CDC) growth charts.^9^ Two case groups comprised: LHW boys aged ≥10 years and LHW girls aged ≥9 years, who met the above criteria. The children in these groups were enrolled in another study that investigated the effect of nutritional supplementation on growth in LHW pre-pubertal boys^10^ and girls (ongoing study).

The control groups comprised healthy, pre-pubertal boys aged ≥10 years, and girls aged ≥9 years, with height and weight ≥25th percentile and a BMI within the 5–85th percentile for age and sex. They were recruited for this study through friends and families of the clinic staff. Exclusion criteria for the study and control groups were height-Z score <−2.5 or BMI Z-score <−3.0; a diagnosis of growth hormone (GH) deficiency or GH treatment; organic causes of growth retardation; and chronic drug treatment that may affect appetite, weight, or growth.

The study was approved by the Institutional Review Board [Rabin Medical Center: RMC 0676-14 and RMC 0002-15]. Written informed consent was obtained from parents prior to enrollment of the case groups, and verbal consent was obtained from the parents of the control groups. The study protocol followed STROBE guidelines.

Participants and their parents completed 3-day food diaries, and sleep, physical activity, quality of life, and socioeconomic status. Control-group participants and their parents also completed a pubertal-stage parent assessment questionnaire.

The outcome measures included anthropometric measures, estimated energy requirement (EER) calculated according to the dietary reference intake (DRI) equations^11^ and assessments of physical activity, sleep, quality of life, and nutritional intake. BMI was calculated as weight in kilograms divided by height in meters squared. Height and BMI values were expressed as Z-scores according to CDC growth charts.^9^ Physical activity, sleep, and quality of life were evaluated by relevant questionnaires;^12,13^ and nutritional assessment by 3-day food diaries.

For the case group, height was measured using a commercial Harpenden stadiometer (Holtain Ltd., Crosswell, United Kingdom) and weight was measured in light clothing using a standard calibrated scale. Parents of the control group were instructed to measure their children’s height and weight at home according to the CDC guidelines^14^ and to report the dates of measurement and the children’s date of birth, for calculating Z-scores.

For the case group, pubertal status was determined by a pediatric endocrinologist. For the control group, pubertal status (pre-pubertal vs. pubertal) was determined according to parent-assessment questionnaires. The questionnaire was drafted by a pediatric endocrinologist at the Institute of Endocrinology and Diabetes at Schneider Children’s Medical Center, and was designed to differentiate between pre-pubertal children (Tanner stage 1) and all other stages of puberty. The questionnaire was also filled by parents of the case-group participants, and its results were comparable with the pubertal status determined by a pediatric endocrinologist.

After undergoing a short, structured course of training by a dietician, the parents and children were asked to record all the children’s food and beverage consumption. The information included the actual time of consumption, and portion sizes consumed over a 3-day period (two weekdays, one weekend) during the week before enrollment. Dietary intake was analyzed using Tzameret v3 (2016) software, developed in collaboration with the Israel CDC and Ministry of Health.

The EER, defined as the average dietary energy requirement predicted to maintain energy balance in healthy individuals, includes growth-associated needs in children. The EER was calculated according to DRI equations in accordance with age, sex, weight, height, physical activity level, and BMI percentile.^11^ The physical activity level (PAL) was based on parent-reported questionnaires on the type and duration of physical activity performed during a regular week. The scores were defined as sedentary (PAL 1.0–1.39), low active (PAL 1.4–1.59), active (PAL 1.6–1.89), and very active (PAL 1.9–2.5).^11^

Sleep habits were evaluated by the Sleep-Schedule Time Questionnaire.^12^ Parents answered questions regarding their children’s sleeping habits. These included the time of awakening, of getting out of bed, of readiness for sleep (turning off lights), time until falling asleep (after turning off lights), and nap times during the day. The total hours of sleep were calculated from the questionnaire, separately for weekdays and weekends.

Health-related quality of life was assessed using the PedsQL 4.0.^13^ This questionnaire was validated for numerous pediatric medical conditions and in healthy youth, aged 2–18 years. The PedsQL Hebrew version was linguistically validated and has been used on Israeli sample populations.^15^

Socioeconomic position (SEP) was calculated by the participants’ home addresses according to the Israel Central Bureau of Statistics’ Characterization and Classification by the Socio-Economic Level of the Population 2015.^16^ The SEP index is an adjusted calculation of 14 variables that measure social and economic levels in the domains of demographics, education, standard of living, and employment.

Data were analyzed by the IBM SPSS software (IBM SPSS Statistics for Windows, Version 27; IBM Corp., Armonk, NY). Continuous variables are presented as means and standard deviations (normal distribution), or as medians and interquartile ranges (skewed distribution); and categorical variables as numbers and percentages. Between-group differences in continuous variables were examined using independent-sample t-tests (normally distributed data) or the Mann–Whitney U-tests (skewed data). Between-group differences in categorical variables were examined using Pearson’s chi-square test. To test the null hypothesis that means or medians of micronutrient intakes were not different from 100% recommended daily allowance (RDA), we used the one-sample t test or one-sample Wilcoxon signed rank test, for variables with normal or skewed distribution, respectively.

We conducted logistic regression multivariate analyses (four models) for associations of being in the case group (LHW), with nutritional and lifestyle parameters. All the models were adjusted for age and SEP. The variables included in each model were determined by the Forward logistic regression method. Model 1 included the % energy intake/EER and macronutrient intake (g/kg body weight [BW]): proteins, fats, and carbohydrates. Model 2 included the intakes of saturated fat and monounsaturated fats (% of total energy intake), sugars (g), and leucine (g). Model 3 included the intakes of micronutrients (% RDA/10): iron, zinc, calcium, vitamin A, and vitamin C. Model 4 included lifestyle parameters, namely sleep during weekdays and the weekend (hours/ day), and PAL (sedentary, low active, active, and very active).

The sample size was calculated using previous data on iron consumption in boys and girls of the same age-range.^17^ According to these data we assumed a difference of 22 mg/day between the case and control groups, and a standard deviation of 23.3 mg/day, with an 80% chance of detecting a significant increase at the 2-sided 5% level. The calculated sample size was at least 18 participants in each group.

## Results

A total of 111 pre-adolescents participated in the study: 32 boys and 31 girls in the case groups, and 24 boys and 24 girls in the control groups. One girl from the case group was excluded from the analysis due to extremely low caloric intake according to the 3-day food diary (average of 371 Kcal/day), probably due to underreporting.

Table 1 presents clinical and demographic characteristics of the participants. The median ages of the boys and girls in the case groups (11.5 and 10.2 years, respectively) were about one year above those of the control groups (10.2 and 9.7 years, respectively) (*P* < 0.001 for both comparisons). As expected from the group eligibility criteria, height-Z, weight-Z, and BMI-Z scores were lower in the case than the control groups (*P* < 0.001). The mean values of mid-parental height (MPH) (cm and Z-scores) were also lower in the case than the control groups (*P* = 0.004 for boys and *P* < 0.001 for girls). Of note, in the case groups, the MPH Z-scores were higher than the children’s height-Z scores (*P* < 0.001 for both boys and girls); while in the control groups, MPH-Z scores were similar to the children’s height-Z scores (*P* > 0.05 for both boys and girls). The differences between MPH-Z score and the children’s height-Z score were significantly higher in the case than the control groups (*P* < 0.001 for both boys and girls). The SEP index scores were above average and similar in the case and the control groups (*P* = 0.569 for boys and *P* = 0.886 for girls).

**Table 1:** Clinical and demographic characteristics of the participants in the case and control groups, according to sex.

	Boys			Girls		
	Case^a^ (*n* = 32)	Control^b^ (*n* = 24)	*P*	Case^a^ (*n* = 31)	Control^b^ (*n* = 24)	*P*
Age (years)	11.5 (11.1, 12.0)	10.2 (10.0, 10.6)	<0.001	10.7 (10.0, 11.3)	9.7 (9.3, 9.8)	<0.001
Height-Z score	−1.7 (−2.0, −1.6)	0.6 (0.2, 0.8)	<0.001	−1.8 (−1.9, −1.6)	0.0 (−0.3, 0.5)	<0.001
Weight-Z score	−2.2 (−2.5, −1.9)	0.2 (−0.2, 0.7)	<0.001	−2.2 (−2.5, −2.0)	−0.2 (−0.7, 0.3)	<0.001
BMI-Z score	−1.6 (−1.9, −1.1)	0.1 (−0.4, 0.6)	<0.001	−1.5 (−1.8, −1.3)	−0.4 (−0.9, 0.2)	<0.001
MPH (cm)	173.2 ± 4.4	177.7 ± 5.1	0.004	160.2 ± 5.4	166.6 ± 4.3	<0.001
MPH-Z score	−0.41 ± 0.60	0.22 ± 0.71	0.004	−0.44 ± 0.83	0.54 ± 0.67	<0.001
MPH-Z minus Height-Z	1.38 ± 0.57	−0.28 ± 0.74	<0.001	1.33 ± 0.76	0.34 ± 0.67	<0.001
SEP Z-score	0.6 (0.5, 1.4)	0.7 (0.2, 1.2)	0.569	0.8 (0.6, 1.3)	1.1 (0.2, 1.4)	0.886

The data are presented as mean ± SD (normal distribution) or median (interquartile range) (skewed distribution). *P* values represent the independent samples t-test or the Mann–Whitney U-test, for variables with normal or skewed distribution, respectively.

*BMI* Body mass index, *MPH* Mid-parental height, *SEP* socioeconomic position.

^a^Height and weight <10th percentile, weight ≤height percentile.

^b^Height and weight ≥25th percentile.

### Macronutrients

Among the boys, the absolute mean energy intake was lower for the case than the control group (*P* = 0.046). However, when adjusted to energy requirements (energy intake/EER), the difference was no longer significant (*P* = 0.194). Among girls, both total energy intake and energy intake/EER were comparable for the case and the control groups (Table 2).

**Table 2:** Nutritional intake of the participants in the case and control groups, according to sex.

	Boys			Girls		
	Case^a^ (*n* = 32)	Control^b^ (*n* = 24)	*P*_1_^c^	Case^a^ (*n* = 31)	Control^b^ (*n* = 24)	*P*_1_^c^
Energy, Kcal/day	1720 ± 284	1897 ± 365	0.046	1360 ± 279	1469 ± 292	0.164
Energy/EER (%)	87.5 ± 16.9	81.4 ± 17.2	0.194	82.2 ± 20.6	77.9 ± 20.6	0.447
Protein (g/day)	63.9 ± 13.9	79.8 ± 15.4	<0.001	52.8 ± 15.0	60.7 ± 15.2	0.059
Protein/weight (g/kg/day)	2.3 ± 0.6	2.3 ± 0.5	0.580	2.2 ± 0.7	2.0 ± 0.5	0.296
Leucine (g/day)	3.7 ± 1.3	4.9 ± 1.4	0.003	3.2 ± 1.2	3.7 ± 1.2	0.113
Leucine/weight (g/kg/day)	0.14 ± 0.05	0.14 ± 0.04	0.866	0.13 ± 0.06	0.12 ± 0.04	0.454
Carbohydrates (g/day)	221.8 ± 44.4	227.5 ± 49.0	0.649	170.4 ± 38.3	177.3 ± 43.0	0.529
Carbohydrates /weight (g/kg/day)	8.1 ± 1.8	6.5 ± 1.6	<0.001	7.0 ± 1.5	5.8 ± 1.4	0.007
Sugar (g/day)	64.8 ± 29.1	70.0 ± 31.6	0.528	47.3 ± 21.8	53.5 ± 20.9	0.292
Sugar/weight (g/kg/day)	2.4 ± 1.0	2.0 ± 1.0	0.191	1.9 ± 0.9	1.8 ± 0.7	0.489
Fat (g/day)	62.7 ± 13.6	70.9 ± 17.6	0.054	49.8 ± 13.8	54.7 ± 11.4	0.164
Fat/weight (g/kg/day)	2.3 ± 0.6	2.0 ± 0.5	0.050	2.0 ± 0.6	1.8 ± 0.4	0.082
Saturated fat (g/day)	21.8 ± 7.0	23.5 ± 7.3	0.382	17.0 ± 5.1	19.6 ± 5.6	0.074
Saturated fat /weight (g/kg/day)	0.80 ± 0.27	0.67 ± 0.22	0.053	0.69 ± 0.21	0.65 ± 0.18	0.358
Mono-unsaturated fat (g/day)	20.8 (17.8, 22.9)	27.3 (19.8, 31.5)	0.004	16.4 (15.0, 22.0)	19.7 (16.8, 23.4)	0.078
Mono-unsaturated fat /weight (g/kg/day)	0.8 (0.6, 0.9)	0.8 (0.6, 0.8)	0.843	0.7 (0.6, 0.9)	0.7 (0.6, 0.7)	0.159
Calcium (mg/day)	489 ± 240	725 ± 310	0.002	488 ± 170	510 ± 197	0.668
RDA (%)	38 ± 18	56 ± 24		37 ± 13	39 ± 15	
*P*_2_^d^	<0.001	<0.001		<0.001	<0.001	
Iron (mg/day)	8.9 (7.2, 10.0)	11.7 (10.5, 15.5)	<0.001	8.1 (7.3, 9.6)	7.3 (6.7, 9.0)	0.263
RDA (%)	112 (91, 125)	146 (132, 194)		101 (91, 120)	92 (83, 113)	
*P*_2_	0.089	<0.001		0.281	0.648	
Zinc (mg/day)	6.7 (5.7, 8.1)	8.5 (7.1, 10.8)	0.004	5.4 (4.1, 6.8)	5.7 (4.9, 6.8)	0.256
RDA (%)	84 (72, 101)	107 (89, 135)		67 (51, 85)	72 (61, 85)	
*P*_2_	0.006	0.153		<0.001	<0.001	
Vitamin A (µg/day)	238 (153, 350)	499 (348, 722)	<0.001	269 (195, 418)	337 (255, 545)	0.069
RDA (%)	40 (26, 58)	83 (58, 120)		45 (32, 70)	56 (43, 91)	
*P*_2_	<0.001	0.376		<0.001	<0.001	
Vitamin C (mg/day)	57 (24, 75)	78 (39, 116)	0.037	37 (22, 77)	64 (30, 98)	0.235
RDA (%)	127 (54, 167)	174 (87, 257)		84 (48, 171)	141 (66, 217)	
*P*_2_	0.112	0.002		0.814	0.067	

The data are presented as mean ±SD (normal distribution) or median (interquartile range) (skewed distribution).

*EER* Estimated energy requirements, *RDA* Recommended dietary allowance.

^a^Height and weight <10th percentile, weight ≤height percentile.

^b^Height and weight ≥25th percentile.

^c^*P*_1_ values represent the independent samples t-test or the Mann–Whitney U-test, for variables with normal or skewed distribution, respectively.

^d^*P*_2_ values represent the one-sample t test or the one-sample Wilcoxon signed rank test, for variables with normal or skewed distribution, to test the null hypothesis that the mean or median is not different from 100% RDA.

Among the boys, for the case compared to the control group, intakes were lower of protein (*P* < 0.001), leucine (*P* = 0.003), and monounsaturated fat (*P* = 0.004). After adjustment to body weight (BW, g/kg), the differences were no longer significant (*P* > 0.05) (Table 2). Among the boys, for the case compared to the control group, the mean intake of carbohydrates/kg BW was higher (*P* < 0.001); and intakes tended to be higher of fat/kg BW (*P* = 0.050) and saturated fat/kg BW (*P* = 0.053). Among the girls, the only significant difference between the case and control groups was higher carbohydrate/kg BW intake in the former (*P* = 0.007) (Table 2). Logistic regression models adjusted to age and SEP index showed similar results as those detailed above (Table 3). According to these models, being in the case groups was associated with higher carbohydrate/kg BW intake. Model 1, analyzed for boys, showed an odds ratio (OR) of 1.8, 95%CI (1.07, 3.02); and analyzed for girls, an OR of 3.02, 95%CI (1.40, 6.53). Model 2 showed that for boys, being in the case group was also associated with lower intakes of monounsaturated fat [OR = 0.66, 95%CI (0.43, 1.01)] and leucine [OR = 0.40, 95%CI (0.18, 0.87)].

**Table 3:** Sex specific logistic regression multivariate models for the associations between being in the case group (low height and weight), and nutritional and lifestyle parameters.

	Adjusted OR^a^	95% CI for OR	*P*
Model 1^b^: Energy and macronutrients			
Boys			
Carbohydrates (g/kg)	1.80	1.07, 3.02	0.026
Girls			
Carbohydrates (g/kg)	3.02	1.40, 6.53	0.005
Model 2^c^: Specific nutrients			
Boys			
Mono-unsaturated fats (% of energy)	0.66	0.43, 1.01	0.055
Leucine (g)	0.40	0.18, 0.87	0.020
Girls			
No variable entered the model			
Model 3 ^d^: Micronutrients			
Boys			
Iron (%RDA/10)	0.82	0.64, 1.05	0.144
Vitamin A (%RDA/10)	0.65	0.44, 0.97	0.037
Girls			
No variable entered the model			
Model 4^e^: Lifestyle parameters			
Boys			
Sleep during weekdays (hours/day)	0.22	0.06, 0.78	0.019
Girls			
Sleep during weekends (hours/day)	0.30	0.10, 0.87	0.027

*RDA* Recommended dietary allowance.

^a^All the models were adjusted for age and socioeconomic status, using the Forward logistic regression method.

^b^Model 1 included the variables: energy (% energy/estimated energy requirements) and macronutrients (g/ kg body weight), namely, proteins, fats, and carbohydrates.

^c^Model 2 included the variables: saturated fats and mono-unsaturated fats (% of total energy), sugars (g), and leucine (g).

^d^Model 3 included micronutrients (% RDA/10), namely, iron, zinc, calcium, vitamin A, and Vitamin C.

^e^Model 4 included lifestyle parameters, namely sleep during weekdays and during the weekend (hours/ day), and physical activity level (sedentary, low active, and active).

### Micronutrients

Among the boys, in the case compared to the control group, the mean intakes were lower of calcium (*P* = 0.002), iron (*P* < 0.001), zinc (*P* = 0.004), vitamin A (*P* < 0.001), and vitamin C (*P* = 0.037). Among the girls, significant differences in micronutrient intake were not observed, between the case and control groups (Table 2). The logistic regression models showed similar results (Table 3). According to model 3, for boys, being in the case group was associated with lower iron and vitamin A intake (as %RDA /10). The ORs were 0.82, 95%CI (0.64, 1.05) and OR = 0.65, 95%CI (0.44, 0.97), respectively. For all the groups, the mean calcium intake was lower than RDA recommendations (*p* < 0.001). For all the groups except the male control group, mean intakes of zinc (*P* < 0.010) and vitamin A (*P* < 0.001) were lower than RDA recommendations (P_2_, Table 2).

### Lifestyle parameters

Among the boys, for the case compared to the control group, the median sleep duration was shorter during weekdays (8.8 vs. 9.5 hours/day, *P* < 0.001). No other differences were found between these two groups, in PAL, and in the quality-of-life questionnaires. Among the girls, such differences were not found between the case and control groups (Table 4). Logistic regression models showed that for boys, being in the case group was associated with shorter sleep duration during weekdays [OR = 0.22, 95%CI (0.06, 0.78)]. For girls, being in the case group was associated with shorter sleep duration during the weekend [OR = 0.30, 95%CI (0.10, 0.87)] (Table 3, model 4).

**Table 4:** Physical activity, sleep patterns and quality of life in the case and control groups, according to the sex of the participants.

	Boys			Girls		
	Case^a^ (*n* = 32)	Control^b^ (*n* = 24)	*P*	Case^a^ (*n* = 31)	Control^b^ (*n* = 24)	*P*
Physical activity, level *n* (%)						
Sedentary	4 (12.5%)	7 (29.2%)	0.297	17 (54.8%)	13 (54.2%)	0.381
Low active	11 (34.4%)	7 (29.2%)		9 (29.0%)	4 (16.7%)	
Active	17 (53.1%)	10 (41.7%)		5 (16.1%)	7 (29.2%)	
Sleep duration (hours/day)						
Weekdays	8.8 (8.5, 9.3)	9.5 (9.2, 10.0)	<0.001	9.3 (9.0, 9.8)	9.5 (9.3, 9.8)	0.193
Weekend	9.8 (8.9, 10.9)	9.5 (8.6, 10.4)	0.234	9.5 (9.3, 10.4)	10.0 (9.5, 10.6)	0.140
Quality of life (Peds QL questionnaire) score						
physical functioning	86 (73, 100)	88 (78, 94)	0.904	88 (81, 97)	88 (75, 97)	0.895
emotional functioning	75 (58, 90)	65 (90, 80)	0.138	70 (60, 85)	60 (50, 80)	0.243
social functioning	98 (81, 100)	85 (75, 100)	0.145	100 (80, 100)	95 (75, 100)	0.403
school functioning	75 (61, 94)	80 (70, 90)	0.246	85 (70, 95)	80 (65, 95)	0.585
Total score	83 (67, 93)	83 (74, 88)	0.918	80 (74, 93)	82 (70, 91)	0.581

The data are presented as number (percent) for categorical data, mean ± SD (normal distribution) or median (interquartile range) (skewed distribution).

*P* values represent the Pearson’s chi-square test for categorical variables; or the independent samples t-test or Mann–Whitney U-test, for numerical variables with normal or skewed distribution, respectively.

^a^Height and weight <10th percentile, weight ≤height percentile.

^b^Height and weight ≥25th percentile.

## Discussion

This study was designed to examine whether the diet and sleep of pre-adolescents who were of relatively short stature and light weight, and significantly shorter than their MPH, is distinctly different from peers of taller stature and weight. The fact that these children were shorter than their MPH may be explained by both genetic (such as delayed maturation) and environmental factors, that underlie the relatively low height and body weight. This otherwise healthy population may be overlooked by caretakers, as height percentile within the norm is commonly assumed to be solely genetically determined.

Our initial decision to divide the cohort according to sex was driven by previously reported sex differences in eating behaviors, nutritional status and micronutrient deficiencies and sleep patterns. This has been shown both in adolescents^18,19^ and in adults,^20^ residing in both low-income countries and in developed countries.^21,22^ The suggested explanations for these sex differences range from physiologic differences, (e.g. body composition), to psychological, cultural and behavioral differences,^20,23^ which affect food choices, sleep patterns and dietary behaviors.

Among the boys in our cohort, both for those who were LHW as well as controls, the macronutrient intake/kg BW and energy intake/EER were adequate. However, intakes of zinc, iron, calcium, vitamin C, and vitamin A were lower in the case group, and did not meet RDA recommendations. Among the girls, both for those who were LHW and for the control group, macronutrient intakes were adequate; and calcium, zinc, and vitamin A intakes were comparable, yet insufficient according to recommendations. The observed insufficient intakes of micronutrients in LHW boys corroborate a number of observational and clinical studies. Specifically, important roles have been described for calcium, zinc, and vitamin A in linear growth.^24^ The nutritional data among the boys in this cohort are consistent with a previous study that was performed by our group, on younger LHW children (aged 3–9 years).^6^ Similar to the current study, energy intake was adequate, and comparable to that of a control group, while micronutrient consumption was inadequate in both boys and girls.

In both LHW boys and girls, energy intake was in accordance with the DRI recommendation, and energy intake/EER was similar to that of the control groups. Therefore, the lower height-Z score in this cohort cannot be explained by insufficient energy intake. However, it may be possible that the measured resting energy expenditure (REE)/kg BW is higher in children with short stature than calculated by DRI equations, as shown previously.^25^ Similar results were found in adolescent boys with constitutional delay of growth and puberty, compared to age-matched controls.^26^ Notably, we did not measure REE, but instead estimated energy needs according to equations. Thus, we cannot rule out the possibility that the calculated EER underestimates the energy required for growth in LHW children, which may be unmet by caloric intake.

The mean intake of carbohydrates/kg BW was higher among LHW boys and girls than among their respective control groups. In boys, the consumption of saturated fat/kg BW was also higher in the LHW than the control group, and the consumption of monounsaturated fat was lower. Taken together with the inadequate consumption of micronutrients, the results may reflect a diet of poorer quality in the LHW groups.^27^ While, among boys, total protein consumption was adequate and similar for both groups, the mean leucine intake was lower for the LHW than the control group. Both in animal models, and in stunted children from developing countries, leucine has been associated with bone elongation and its deficiency with stunting.^28,29^ Our results support the growing evidence that the quality as well as the quantity of protein is important for linear growth and eventual final height.^30^

Among boys, we found a shorter duration of sleep on weekdays for the LHW than the control group. Among girls, we found an association of less sleep during weekends (model 4) with being in the LHW group. In a previous study by our group, improved nutritional intake was associated with improved sleep patterns among LHW children aged 3–9 years.^31^ However, it is unclear whether less sleep is associated with poorer growth,^32^ and admittedly this study was not designed to examine the causality of these results.

In the current study, sex differences in nutrient deficiencies and sleep patterns were found as expected. A recent systematic review (34 studies, n = 1694) studied the different motivations for food choices in adolescents. The authors found that girls placed a greater emphasis on external motivators (e.g., eating healthier, altering dietary habits around boys, social norms of thinness) in shaping their dietary choices, while boys were more driven by internal motivators (e.g., gaining autonomy, eating for pleasure, and improving physical performance).^23^ We can therefore assume that sex or gender related differences in behavior are the major cause for the differences found in our study population, but this needs to be examined in future studies.

While the majority of studies on childhood nutrition focus on early childhood, there is a paucity of data on interactions between nutrition and growth in older children and adolescents. Yet, several studies have shown that nutritional deficits that persist throughout childhood affect final height, even in affluent societies. For example, uncompensated undernutrition in children with milk allergy was shown to result in shorter adult height.^33^ In another study, consumption of dairy products during adolescence was associated with taller stature.^34^ Undernutrition in girls with anorexia nervosa during adolescence was reported to have an irreversible impact on growth.^35^ Moreover, healthier eating habits among adolescents were found to be associated with taller stature, while unhealthy eating habits were associated with shorter stature.^27^ These studies highlight the importance of nutritional surveillance and intervention in adolescence, and not only in younger children. Notably, for children within this age range whose diet is energy sufficient, the quality of nutrition may be overlooked by caregivers.

This study has several limitations. An important limitation in the study design is the self-reporting of intake in all groups, and of height, weight and pubertal stage in the control groups. Although self-reported intake is a well-established method for assessing intake,^36,37^ results should be interpreted accordingly. We are aware that food records underestimate true intakes of energy and protein by 4–37%.^38,39^ However, this population of children with low to normal BMI-Z scores is not one that should be prone to underreporting energy intake.^40^ Regarding the self-reported height, weight, and pubertal status in the control group, special attention was paid to enhance the representativeness of the group, and to ensure the most accurate measurements. We have previously demonstrated that home measurements of weight and height, when conducted with proper guidance from our institute using CDC protocols, are highly reliable.^41^ Pubertal status in the control group was determined using parent-assessment questionnaires, which were developed by a pediatric endocrinologist from our Institute and designed to distinguish pre-pubertal children (Tanner stage 1) from those at other stages of puberty. Notably, the same questionnaire was administered to parents of the case group participants, with results closely aligning with pubertal assessments made by a pediatric endocrinologist, providing additional confidence in the reliability of the self-reported data.

Regrettably, the mean age of the children in the case groups was higher than the mean age of the children in the control groups. This can be explained by the study inclusion criterion of pre-puberty (Tanner 1). In light of the negative association between BMI and timing of puberty, recruiting age-matched controls to our lean cohort who were still pre-pubertal was challenging. In order to mitigate this bias, all the predictive models in the study were adjusted for age.

Finally, our study was designed as a case-control study, which cannot prove causality between environmental exposures and outcomes. However, it can provide a detailed description of the current differences between the comparison groups.

In conclusion, our results strengthen previous findings of poorer diet quality among children with short stature, perhaps raising a question regarding the impact of suboptimal nutrition in the lower percentiles of normal growth. Nutritional surveillance and intervention may be beneficial to optimize nutrition and sleep quality in LHW pre-adolescents, especially boys. Further studies are needed to examine the effects of nutrition on growth in otherwise healthy older children with relatively low anthropometric indices.

## Data Availability

The datasets generated during and/or analyzed during the current study are available from the corresponding author on reasonable request.
